# Perspectives of Patients and Providers on Chronic Pain Assessment in Neurofibromatosis Type 1 (NF1): A Qualitative Study

**DOI:** 10.7759/cureus.100193

**Published:** 2025-12-27

**Authors:** Lauretta Grau, William A Zempsky, Staci Martin, Kaitlyn Larkin, Frank D Buono

**Affiliations:** 1 Epidemiology, Yale School of Public Health, New Haven, USA; 2 Pain and Palliative Medicine, Connecticut Children’s Hospital, Hartford, USA; 3 Pediatric Oncology Branch, National Cancer Institute, Bethesda, USA; 4 Psychology, Northern Illinois University, Dekalb, USA; 5 Psychiatry, Yale School of Medicine, New Haven, USA

**Keywords:** chronic pain, neurofibromatosis 1, pain measurement, patient reported outcome measures, qualitative research

## Abstract

Background and objective

Neurofibromatosis type 1 (NF1) typically presents with physical symptoms, neurocognitive impairments, and chronic pain (CP). Existing pain measures, originally developed for cancer pain or other diseases, do not address the specific healthcare needs of NF1 patients. Hence, this study aimed to examine how individuals with NF1-associated CP and their providers characterize the physical and emotional experiences of pain, as well as their perceived needs in assessing NF1-associated CP.

Methods

We performed a qualitative study, employing thematic analysis of data collected from six focus groups with 37 patients with NF1 and 16 one-on-one interviews with clinicians involved in NF1 care

Results

We identified three themes: Describing the Pain Experience, Treating and Dealing With NF1-Associated CP, and Attitudes About Current Pain Measures. Participants perceived certain shortcomings in current pain measures, emphasizing that pain tolerance, adaptation of goals and activities, and the presence of multiple concurrent pain sites are intrinsic to NF1. Patients expressed frustration with the need to quantify a highly subjective and individualized experience using existing measures. Similarly, providers reported frustration that current tools inadequately capture the impact of chronic pain on patients’ daily lives, with some preferring more open, conversational approaches to pain assessment.

Conclusions

The data indicated that pain duration, frequency, regularity, and quality need to be clearly defined and operationalized for patients and providers to similarly understand these domains. Incorporating open-ended items that allow patients to describe their pain in their own words or through images may enhance patients’ sense of being understood by their providers. There is a clear need for a standardized pain assessment tool that specifically captures the pain experiences of individuals with NF1.

## Introduction

Neurofibromatosis type 1 (NF1) is an autosomal dominant disease that presents with physiological symptoms (e.g., nodules, tumors), neurobehavioral issues, and chronic pain (CP) [1]. Nearly 70% of individuals with NF1 have CP associated with tumors and often not localized to a structural lesion, thus presenting treatment challenges for patients and their medical caregivers [2,3]. Their CP can interfere with quality of life, including sleep problems, maintaining employment and social relationships, and feelings of isolation [4]. Individuals with NF1-associated CP may also encounter barriers to accessing pain-focused care, including limited service availability in many regions, long wait times for appointments, a shortage of qualified professionals, particularly in non-urban areas, patient-related constraints such as transportation or work and family responsibilities, and healthcare system obstacles, including reimbursement issues and out-of-pocket treatment costs [5-7].

Existing and validated assessments such as the Brief Pain Inventory-Short Form (BPI-SF) [8] or Pain Interference Index (PII) [9] were originally developed for cancer pain or other diseases [10]. While previously published NF1 research used these assessments [11-14], some consider NF1-associated CP distinct from other conditions [15,16]. A systematic review evaluated pain assessment tools but focused solely on pain severity and interference, without addressing psychosocial characteristics or other potential features or correlates of NF1-associated CP [10]. There is a need for an assessment instrument that more fully captures the unique aspects of NF1-associated CP [17]. In this context, qualitative interviews represent a critical first step in measure development [18], as they help elucidate patient and clinician perspectives on NF1-associated CP and the perceived usefulness of existing assessment tools.

To our knowledge, only generalized pain measures have been recommended for NF clinical trials [10], and no validated pain measure specific to NF currently exists. Therefore, as part of our broader effort to develop an NF1-specific pain assessment, this study sought to analyze how individuals with NF1-associated CP and their healthcare providers describe the physical and emotional experiences of pain, as well as the perceived needs in assessing NF1-associated CP. Additionally, any NF1-associated CP measure should include clearly defined descriptions and definitions of pain domains and functional impact. Providing this level of clarity may improve the standardization of pain assessment for NF1-associated CP among healthcare providers and researchers and support more effective treatment and management for individuals with NF1.

## Materials and methods

The methods and findings for the current study have not been previously presented or published. The study was conducted between June 1, 2021, and June 30, 2023. The study was based in New Haven, Connecticut, but data collection occurred remotely; therefore, participants resided in multiple locations across the United States.

Recruitment and data collection procedures

Recruitment of NF1 patients, referred to hereafter as the “patient group,” involved sending a mass email announcement through the Children’s Tumor Foundation (CTF) registry (https://www.ctf.org/nf-registry/) that briefly described the study. Membership in the registry requires self-report of a formal NF diagnosis. Individuals interested in participating completed an online screening survey administered via Qualtrics (www.qualtrics.com) that collected demographic information including age and gender, contact information, and responses to the Brief Pain Inventory Short Form (BPI-SF) [8], Patient Health Questionnaire (PHQ) [19], Structured Clinical Interview for DSM Self Report version (SCID-SR) [20], and Generalized Anxiety Disorder 7 Item version (GAD-7) [21]. The PHQ-9 and GAD-7 are copyrighted by Pfizer Inc. and are freely available for research and clinical use without requiring special permissions. The BPI-SF and SCID-SR were used with written permission from their respective copyright holders.

Inclusion criteria for the patient group included: (1) age 18 to 75 years, (2) membership in the NF Registry, (3) a pain severity aggregate score of ≥3 on the BPI-SF [8] for pain experienced in the previous 2 weeks [10], (4) agreement to allow audio recording, and (5) current residence in the USA. Exclusion criteria were: (1) evidence of moderate to severe depression or anxiety on the PHQ or GAD-7, or (2) evidence of psychosis or schizophrenia on the SCID-SR [20]. Members of the research team (FB, KL) reviewed the clinical screening measures, and all eligible individuals were provided with a list of focus group discussion (FGD) dates and times from which they could choose.

Recruitment of NF1 clinicians (to be referred to henceforward as the “provider group”) occurred via direct emails to a purposive sample of 100 providers from the CTF “Find a Doctor” page (https://www.ctf.org/find-a-doctor/). Although the interviews were originally proposed as FGDs, providers’ limited availability rendered it unfeasible to convene these sessions promptly. We therefore conducted individual interviews with the providers instead. Inclusion criteria for the provider group were: (1) provides healthcare to patients with NF1 (e.g., physician, nurse practitioner, speech pathologist, or psychologist) and (2) agreement to be audio-recorded. Data collected included professional/job title, years in practice, and gender, and was obtained at the beginning of each interview.

Before beginning the interview sessions, participants in both groups received a brief description of the study, procedures, and risks and benefits, and were informed that participation was voluntary. They then provided verbal informed consent. Participants in the FGDs also agreed to keep the content of the discussions confidential. The Yale University Institutional Review Board reviewed and approved the study and all associated materials. Two members of the research team (FB, KL) with prior training in semi-structured interviews jointly facilitated each FGD session, while the senior author conducted all individual interviews. The FGD sessions each lasted approximately one hour, and the individual interviews each lasted approximately 30 minutes. All sessions were audio-recorded, transcribed verbatim, and proofread before analysis.

The interview guides for both participant groups were created specifically for this study (see Appendix) and did not include questions from previous guides or semi-structured interviews. The FGD guide for the patient group covered three domains. The first domain assessed the pain experience. It asked patients to provide specific terms they commonly use to describe their pain, identify other factors they believe are associated with their CP, describe any attempts to systematically track their pain, and explain how NF1-associated CP interfered with their daily lives. The second domain explored how CP could impact other aspects of patients’ lives, such as emotional responses to pain and coping strategies. The final domain gathered patients’ opinions on the usefulness of current pain measures and which elements of pain were most important to monitor.

The provider interview guide included four domains, with the first three aligned with those of the FGD guide. The first domain focused on providers’ perspectives regarding their patients’ experiences of pain. The second domain asked providers how they believed pain was related to other aspects of their patients’ lives. The third domain sought opinions on current pain measures, the adequacy of pain terminology, and how patients track their pain. The fourth domain asked providers whether NF1-associated CP has unique characteristics and the importance of assessing other non-pain signs or symptoms as part of NF1 pain management. At the conclusion of each FGD or individual interview, participants were reminded about confidentiality and the overall goal of the study, thanked for their time, and sent Amazon gift cards ($25 US for the patient group and $50 US for the provider group).

Data analysis

The data analysis team (FB, KL, LG) met regularly to create the codebook and identify potential emergent themes. All team members were experienced qualitative researchers. Interview sessions and data analysis occurred simultaneously and iteratively until the team concluded that data saturation had been achieved (that is, no new information emerged in subsequent interviews) [22-24]. Starting with an initial set of codes based on the domains in the Interview Guide, the coding team independently coded all transcripts and then reviewed them collectively. The codes were developed inductively, reviewed iteratively, and revised based on team discussions of additional transcripts. Any discrepancies in coding were discussed and resolved through consensus.

The coded transcripts were entered into ATLAS.ti (Version 9; Kansas City, MO). Using thematic analysis [25,26], the team examined patterns across the full data set to develop a final, concise set of themes. The analysis also considered any negative instances in which data did not align with the themes or where responses to specific questions were absent. Descriptive statistics were calculated to summarize the study sample.

## Results

Description of the study sample

As shown in Table 1, six FGDs (n = 37) involving NF1 patients were conducted between September 2022 and June 2023. The majority of participants were female, with a mean age of 44.6 years (SD, 16.2; range, 18 - 64 years). Participants estimated their average pain during the previous two weeks to be moderate to severe, but lower at the time of assessment. As previously noted, those with moderate to severe depression or severe anxiety (i.e., scores of >15 on either measure) were excluded. The mean score for depression on the PHQ was 12.1 (SD, 2.3; range, 0 - 14), and the mean score for anxiety on the GAD-7 was 10.7 (SD, 1.1; range, 0 - 14).

**Table 1:** Description of the study sample BPI-SF: Brief Pain Inventory - Short Form; PHQ: Patient Health Questionnaire; GAD-7: General Anxiety Disorder - 7; SD: standard deviation

Variables	Values
	N (%)
Gender	
Male	11 (29.7)
Female	26 (70.3)
	Mean (SD)
Age (years)	44.1 (16.2)
BPI-SF	
Past 2 weeks	4.8 (1.8)
At the time of the interview	3.7 (2.9)
PHQ	12.1 (2.3)
GAD-7	10.7 (1.1)

The 16 individual interviews with clinicians were conducted between February and May 2022. The sample included one advanced nurse practitioner, nine physicians (from neurology, hematology, oncology, pediatrics, or psychiatry; two held MD/PhD degrees), and four participants with doctoral degrees in psychology or speech and language pathology. All but one participant were university affiliates at U.S. institutions. The remaining participant was a clinical psychologist working in private practice.

Opinions about the possible uniqueness of NF1-related pain

To set the context for examining perceptions about NF1-associated CP (Table 2), providers discussed whether it differed from pain experienced by patients without NF1. Almost all providers had opinions. Many thought there was a unique aspect to NF1-associated CP and focused primarily on its etiology or onset (e.g., “accidental trauma”) or on the observable physical disfigurement associated with the pain. 

**Table 2:** Setting the context - quotes NF1: neurofibromatosis type 1; CP: chronic pain

Providers' Opinions about the possible uniqueness of NF1-related CP	Quotes
NF1 pain is unique	"I think that where the source of the pain and how it comes about, I think, can be unique from other non-NF types of pain." (Provider 6, PhD, neuropsychologist)
	"I do think there is something unique about the way patients with NF1 either experience pain, perceive pain, or how pain is produced." (Provider 10, MD, neurologist)
NF1 pain is not unique	"I think insofar as the tumors involved nerves, they are unique. But the pain itself and pain from cancer, pain from growths that are not neurofibromas, no, I don’t think that’s unique to NF1." (Provider 14, MD, pediatrician)
	“I don't think there's any word that there's not also someone without NF1 has experienced a similar kind of type of pain." (Provider 16, PhD, psychologist)

Others were more equivocal, noting that the painful sensations were not unique but could have “unique aspects” for the NF1 patient. They thought that all pain is described similarly by NF1 patients and nonpatients (i.e., that pain is pain is pain). 

Thematic analysis

We identified three themes across the data from both participant groups: (1) Describing the Physical CP Experience, (2) Treating and Dealing With CP, and (3) Attitudes About Current Pain Measures. The first theme identified the specific terms that participants used to describe their NF1-associated CP. The second theme focused on how both groups believed that patients coped with NF1-associated CP and the potential effectiveness of its treatment. The third theme centered on opinions about the utility, strengths, and weaknesses of current pain measures. 

Theme 1: Describing the Physical CP Experience

It was important to initially understand how patients and providers typically described NF1-associated CP. Patients and providers most often identified a pain episode using a combination of descriptors that could be grouped into six sub-themes: the Onset sub-theme focused on what triggered a pain event; the Duration sub-theme addressed how long a single pain episode lasted; the Sensation sub-theme included descriptions of how CP felt to the patient; the Severity sub-theme focused on the magnitude of pain, independent of episode duration; the Location sub-theme identified the specific anatomical site of the pain; and the Movement sub-theme addressed whether the pain traveled from one location to another.

Both participant groups were generally consistent in identifying what could trigger pain onset (Table 3). While providers seemed to primarily refer to “some sort of tactile stimulation” or “any strenuous activities,” patients noted several other pain triggers. These included “pounding” noises that triggered migraines, positional pain (e.g., when bending, twisting, reaching), or not being mindful when dressing.

**Table 3:** Quotes for theme 1: Describing the Physical CP Experience CP: chronic pain

Sub-themes	Quotes
Onset sub-theme	[Pain can be] "instigated by mild trauma, so bumping into something." (Provider 10, MD, neurologist)
	“And anything, like pounding, anything like that, it would cause it [pain onset].” (Patient ID? Sex?)
	"…if somebody snaps a towel at me, it seems to hurt more if they hit one of my tumors than if they don't." (Patient 4, male)
Sensation sub-theme - use of similes	“…like I’m actually sitting on a knot.” (Patient 2, female)
	“…like stepping stones grinding against each other.” (Patient 14, female)
	“…like someone was literally peeling the skin off my legs.” (Patient 35, female)
Severity sub-theme - limitations of trying to quantify pain	“And when I hear someone tell me right now, ‘I’m having a 10 out of 10 pain,’ but yet you’re talking to me like nothing is happening…I would’ve said it’s inaccurate to say 10 out of 10 because clearly you’re not crying on the floor with pain.” (Provider 3, MD, neurologist/oncologist)
	"I think that trying to get details from a patient is more helpful than just the 1-to-10 scale." (Provider 11, MD, PhD, oncologist)
	“I can use each patient as their own control, and I can determine if an intervention is working.” (Provider 7, MD, neurologist, psychiatrist)
Severity sub-theme - subjectiveness	“I actually hate the “on a scale from 1 to 10, how are you feeling, how bad is your pain?” When it’s compared to what?...they ask it on everything, and I am not a fan of that in any situation.” (Patient 7, female)
	“I'm not a big fan of a scale of 1 to 10 because that's so subjective.” (Patient 26, male)
Severity sub-theme - patients prefer verbal descriptors but use numbers	“It’s not like an 8 or a 9, but it’s always like I’d say a 3, steady throughout the day.” (Patient 28, female)

The terms used to describe pain duration were limited and consistent across both groups. The individual terms clustered into three different groups: (1) permanent, (2) fixed duration, and (3) intermittent. Commonly used words that described permanent pain included “persistent,” “constant,” “continuous,” and “unremitting.” Words describing pain of fixed duration included “lasting for days” (for migraines), “daily,” or occurring regularly. Lastly, “intermittent” was the most common descriptor of duration and included words such as “fleeting,” “temporary,” and “not long-lasting.”

Both groups used the same words to describe pain sensation. The three most common were “burning” (or less often “on fire” or “searing”), “stabbing,” and “sharp.” Although both groups also mentioned “aching” or “achy,” providers were more apt to use these descriptors than patients. Providers also described pain sensation as “shock-like” or “electric,” whereas patients described a similar sensation as “pins and needles”; both groups used the word “tingling.” Similarly, both groups used the words “itching,” “dull,” and “throbbing,” but patients also tended to describe the latter as “pounding” or “pulsating.” Infrequently used descriptors included “numbness,” used by a few providers, and “pressure,” “heaviness,” and “tight,” used by some patients. One unique aspect was patients’ creative use of similes to describe their pain sensation (Table 3). This creative tendency was supported by providers’ observations that some patients went so far as to draw a depiction of their pain sensation.

Both groups discussed the challenge of quantifying pain severity (i.e., on a 0 to 10 scale) and acknowledged the limitations of this approach (Table 3). Some providers felt its use was more appropriate for patients with “a focal point of pain,” but less useful when trying to average the severity of “fluctuating pain.” Other providers stated that patients inflated their scores to “capture how much it’s impacting them,” and preferred instead to talk with patients about their pain rather than review the numeric ratings that appear on most pain measures. Providers believed numeric scales were primarily valuable when monitoring an individual’s progress in pain management over time. Patients complained that the severity scales did not help their providers understand the nature of their CP, and several providers agreed, noting that ratings on these scales tended to be highly subjective and idiosyncratic (Table 3).

Patients more often used verbal descriptors (e.g., “immobilizing,” “mild,” “excruciating,” “intense”) to describe their pain severity. However, somewhat ironically, patients also used numbers during the FGDs to describe their pain, suggesting that either they have adopted this approach despite not liking quantitative ratings or that the shared language between patients and providers is limited when discussing pain severity (Table 3). Patients seemed equally comfortable using verbal descriptors, numbers, or similes. For example, one participant described her pain as “always a 2 or a 3 on that stupid, idiotic pain scale” and later as “usually at a low level like white noise, but sometimes it moves to the level of a scream.” Patients may also have a poor understanding of the 0 to 10 metric, as one participant described her pain as “like a 15.” Using a number beyond the specified range again suggests a limited shared language, with patients believing that existing pain scales poorly convey to providers the magnitude of pain experienced.

Given that the Location sub-theme sought to describe an anatomical region, it was not surprising that the terms used were straightforward and consistent between the two groups. The major difference was that providers tended to use more global terms (e.g., back, neck, limbs, headaches), whereas patients described more specific locations (e.g., jawline, ear, ankles, “ring and pinky fingers,”),” suggesting their need to pinpoint the precise location of the pain. Few terms were used to describe the Movement of pain sub-theme, and both groups primarily used the same two terms: “radiating” or “shooting.” Only one patient spoke about their pain as “traveling” from one location to another.

Theme 2: Treating and Dealing With CP 

In addition to describing their NF1-associated CP, the second theme concerned how NF1-associated CP affected other parts of patients’ lives; it included three sub-themes: (1) Physical and Emotional Ramifications of NF1-associated CP, (2) Stoic Attitude, and (3) Frustration with Pain Treatment. 

Perhaps the most frequently discussed sub-theme was the Physical and Emotional Ramifications of NF1-Associated Pain. It focused on how physical limitations compromised patients’ ability to function and, in turn, their mental health and quality of life (Table 4). Both groups noted that NF1-associated CP made it difficult to concentrate, sleep, or maintain balance. Other physical responses to pain included sensitivity to sound or light and limited use of some parts of the body. Providers were more likely to speak broadly about how NF1-associated CP affected patients’ lives, whereas patients identified more specific physical issues (e.g., nausea, heavy sweating, slurred speech, double vision).

**Table 4:** Quotes for theme 2: Treating and Dealing With Chronic Pain

Sub-themes	Quotes
Physical and Emotional Ramifications of CP	"…if it's interfering with their life, they're inherently going to have a lower quality of life" (Provider 9, PhD, speech and language pathologist)
	[The pain is] "distracting from everything I do throughout the day" (Patient 8, female)
	"It’s like when you’re just in so much pain that you just can’t...meet your basic needs or get out of bed." (Patient 35, female)
	"…stress, depression, anxiety, all those things only amplify pain." (Provider 1, MD, pediatric neurologist)
	"It’s not being able to emotionally or physically be with my daughter" (Patient 10, female)
	"It takes me down a path sometimes of feeling sorry for myself" (Patient 28, female)
Stoicism	"I’ve resigned myself to just being in pain all the time and trying to cope with it to the best of my own ability" (Patient 3, female)
	"[pain is] just a part of life at this point…I try to have a positive attitude about it." (Patient 13, female)
	"I’ve developed such a high pain tolerance that I don’t know when my body is in trouble" (Patient 10, female)
Frustration With Pain Treatment	[It is] "very difficult to sort of match what patients rate their pain as to other markers that clinicians really look at to just try to sort of corroborate or get the full sort of picture." (Provider 4, MD, hematology/oncology)
	“I’ve just kind of accepted that there’s nothing that can be done about it [treating the pain]” (Patient 13, female)

The vast majority of the patient group spoke of living in constant pain, and, despite compromised physical or emotional functioning, the most common coping strategy was to adopt a Stoic Attitude (the second sub-theme) to accomplish daily responsibilities and maintain an acceptable quality of life (Table 4). The harsh reality for most was that they experienced relentless pain, described as “your normal-but it’s not normal.”

NF1-associated CP is often poorly managed, despite providers’ best efforts. Hence, many patients have developed high pain tolerance, noting that “We just push on” and "I can't give in or I would give up." One participant had a broken arm for a month before being diagnosed and noted that “[the pain] was a little bit worse than usual, but it never dawned on me that my arm could have actually been broken." In dismissing their pain, another patient’s dislocated hip went untreated for several days. Nonetheless, few in the patient group were dissatisfied with their NF1 care. Both groups expressed Frustration with Pain Treatment, the third sub-theme (Table 4). Providers noted that CP management in general “was very challenging,” with “not much out there for the NF [patient].” For example, current medications have no effect on NF1-associated migraines.

Theme 3: Attitudes about Current Pain Measures

The third theme concerned opinions about the quality or value of current pain measures when addressing NF1-associated CP. Both groups generally acknowledged several inadequacies in the current pain measures (Table 5). Providers complained that there are no standardized pain measures for NF1 care, although it also seemed that the NRS-11 [27] (a non-specific numeric scale) was commonly used in the field. They also noted that most pain measures have not been validated for different cultures or subcultures, and the number of versions in other languages is limited. One provider noted the difficulty in matching patients’ pain ratings to other pain markers. Another commented on the “great deal of subjectivity in pain,” and another regretted that most (if not all) pain measures do not assess whether the pain resulted from physical or emotional provocation. According to providers, pain measures essentially offer an incomplete picture of limited clinical value.

**Table 5:** Quotes for Theme 3: Attitudes about Current Pain Measures

Sub-themes	Quotes
Provider dissatisfaction	"…pain quality, the temporality, the triggers. I think in general people want more space to talk about that with their physician and ways to feel like it's being tracked and managed rather than just saying, "My pain is a 4. My pain is a 2." (Provider 16, PhD, psychologist)
	"…good for a snapshot, but I – it's not at all comprehensive." (Provider 8, MD, oncologist)
Patient dissatisfaction	“…it's just a lookback…the fact that [the measures are] talking about the past and I'm just having trouble sometimes thinking about how that correlate[s] to the present and my future” (Patient 5, male)
	“…everyone has a different view of what an 8 is or what a 10 is.” (Patient 12, female)

The patient group also appeared dissatisfied with the current pain measures (Table 5). They viewed the inherent subjectivity of numeric measures as lacking clarity - even when such measures attempted to define various points along the scale. Having no standard comparator (e.g., comparing it to post-amputation pain or a mild headache) was another limitation of current measures. Some patients favored using verbal descriptors. Others were confused about how to measure “overall pain” when they had multiple areas of pain, each with varying severity. Only one patient was satisfied with using a numeric scale, largely because it reassured her when her provider’s ratings agreed with hers. 

## Discussion

To our knowledge, this study is the first to examine the perceived adequacy and utility of current pain measures in characterizing NF1-associated CP from the perspectives of patients with NF1 and their healthcare providers. It identified the terms and formats that participants wished to be included in an NF1-specific pain measure. The knowledge gained in this study can be used to create a structured survey for use in a larger, randomized clinical study of NF1 patients and providers as the next step in the development of that NF1-specific pain measure. 

The patient group generally disliked numeric scales and expressed frustration at trying to quantify something as subjective and individualized as the experience of pain. Additionally, patients wished they could either use verbal descriptors or compare their pain with a standard reference, a novel finding of our study that adds to the literature on the use of descriptors in pain measures [28,29]. Providers seemed sensitive to the use of verbal descriptors and were likewise frustrated with the limitations of existing pain measures. They wished for a comprehensive pain measure that could be used consistently by all NF1 providers and validated across different subcultures. Short of that, some providers favored having direct conversations with their patients rather than relying on a measure. They also had mixed opinions about the potential uniqueness of NF1-associated CP. Nonetheless, there remains a need to improve alignment between patients’ and providers’ understanding of how best to assess NF1-associated CP and to measure it more effectively and consistently within and across clinical practices and research studies.

The goal of pain measures is for patients and providers to share a common understanding of the patients’ pain experience, and, in fact, many aspects of that experience are similarly understood by both groups. However, the study revealed other aspects of that experience for which no shared vocabulary exists. This may frustrate patients and leave providers with an incomplete understanding of the situation. The lack of shared vocabulary was most apparent for onset, sensation, and severity. The data suggest a need to expand the vocabulary to include patients’ descriptors of what can provoke a pain response. Further, patients often relied on similes to describe their pain, viewing the available descriptors as limited. Pain domains need to be clearly defined and operationalized so that patients and providers understand them in the same manner. Providing examples with definitions can clarify what information is being sought. Options to localize pain in finer detail on body diagrams, mark multiple pain locations, and include open-ended items (where similes can be used) may help patients feel better understood by their providers. These findings are consistent with existing CP research for other diseases [30,31].

Both groups agreed that the existing pain measures did not adequately address how NF1-associated CP could affect patients’ functioning and quality of life. To our knowledge, this is the first study to document that coping with and treating NF1-associated CP remains an ongoing challenge for patients. Their stoic attitude and high pain tolerance often placed them at increased risk for ignoring medical problems unrelated to their NF1 pain. Yet, as frustrating as it was not to be able to adequately address their CP, patients spoke positively about their providers and acknowledged the difficulty in managing NF1-associated CP.

NF1-associated CP can also affect mental health (e.g., increased anxiety, thoughts of self-harm) and cognitive function [29,32,33]. Existing quality of life measures tailored for NF1 (e.g., PedsQL, Skindex) only include CP as a sub-factor and focus solely on assessing pain severity [34]. Therefore, there appears to be a need to assess NF1-associated CP in greater detail to understand how it may affect mental and physical health.

This study has several limitations. While it sought to gain a detailed understanding of NF1 patients’ and providers’ opinions about current pain measures, the generalizability of the study findings may be limited by the primarily White representation within the study sample. Individuals from racial or ethnic minority populations may differ in their views about the current pain measures. Future studies should seek specific input regarding NF1-associated CP from underrepresented populations. Similarly, individuals with mild pain severity were excluded from the study and may use different terms to describe their pain or hold different opinions about the current pain measures, further limiting generalizability. Nonetheless, data saturation was reached, and we can assume that the most salient issues have been identified, supporting the value of the study findings.

## Conclusions

The current study is the first to provide an understanding of NF1-associated CP from the perspectives of both patients and their clinical providers. The findings suggest that existing CP assessments may not adequately address the treatment needs of patients with NF1 by omitting important information about their CP experience, putting patients at risk of receiving suboptimal care. The findings further suggest that an NF1-specific pain measure is needed that can be used by providers who may not be experts in NF1-associated CP. Therefore, the future measure should clearly define and operationalize the various pain domains, allow for documenting multiple pain locations along with their associated duration, frequency, regularity, and quality, and include an open-ended option for patients to add verbal descriptors or pictures of their CP, as numeric scales were not always considered sufficiently informative.

## Appendices

Appendix 

**Table 6:** Interview guides in tabular format

Readability for Patient Section of Interview Guide: 6.5. Thanks for taking the time to participate in this study! The information we get from you today will help us develop and validate a pain assessment for individuals with NF1. We want to hear from you because you are the individuals living with NF1 and experiencing symptoms. The goal of the study is to create a pain assessment for people with NF1, so that healthcare workers, researchers, and people with NF1 can accurately describe and measure pain symptoms. We are going to ask you to talk about your pain caused by NF1. Specifically, we are looking at two different types of pain symptoms that can come from NF1: One that is caused by migraines due to NF1, and one that is caused by tumor pain due to NF1. There will also be an opportunity to discuss any other types of pain caused by NF1. Each is important, and we will ask you to describe the type of pain you have. As a reminder, this session will be audio-recorded to help us when proofreading the text copy of this session. We may also take notes to help us remember important points raised that we would want to follow up on. I’d like to stress that the potential benefits of creating an NF1-focused pain measure will only be as good as the information we collect, so it’s important for our discussion to be as open and honest as possible. This will help us understand what we will need to do to create a useful NF1-focused pain measure. Do you have any questions before we start? I am now going to start recording the session.
1) DOMAIN 1: Assessing NF1 pain experience. Pain is an unpleasant sensation that can affect multiple areas of the body and one’s ability to function. The emotions associated with that pain can also affect mood and stress levels. To start, we want to understand your experience with NF1 pain.
What words would you use to describe your NF1 pain?
Burning, stabbing, throbbing
What are your thoughts on the importance of using categories for your NF1 pain symptoms?
What other things do you notice happening when you’re in pain
Fast heart rate or sweating
Emotional changes? Changes in function, e.g., ability to move, work, or complete daily living activities?
Do you track your own NF1 pain, and if so, how?
Can you tell me how you measure your pain symptoms?
What do you track?
Length, frequency, inter-response time, and cumulation of all?
How does NF1 pain impact your life?
Does it limit your ability to complete day-to-day tasks?
Quality of Life
Physical functioning
Range of Motion due to tumor vs pain
Pain Behavior
2) DOMAIN 2: How can NF1-associated chronic pain can after other aspects of your life? In this last section, we’d like to understand the impact of your NF1 pain and how it might affect you in other ways beyond just the pain sensation.
How useful are the current pain measurements when it comes to your pain needs?
What makes them useful?
What needs to be added to make them more useful?
How useful do you think the current pain measurements are to your provider when treating your NF1 pain?
What should your doctor know about your pain?
Do they ask about your pain, mood, or sleep?
Which feature is most important to monitor your NF1 pain symptoms?
Severity
Interference
Quality of life
Normal role functioning
Physical functioning
Emotional wellbeing
DOMAIN 3: Opinions about currently available pain measures. In this next section, we want to hear your thoughts on how NF1 pain symptoms are currently measured and how those measures can be improved.
a. What do you think of the current assessments for NF1-associated pain?
Are there merits of the current assessments in your opinion?
If so, what are they?
Are there any demerits of the current assessments in your opinion?
If so, what are they?
What would you like to have measured for pain that is not being measured for pain?
How well do these current assessments evaluate change in NF1-associated pain?
How frequently are these measures needed to get a picture of pain over time?
Is there an impact (normal vs elevated?
b. Beyond measuring the physical manifestations of pain, how important is it to understand or measure other NF1 manifestations of pain (e.g., mood, sleep, executive function)?
What is it that makes measuring these covariates important to you in your practice?
How do you cope with your pain?
What kinds of things do you do to cope with your pain?
What strategies have you used to cope with your pain?
How does your NF1 pain make you respond emotionally?
Discuss the impact of pain on your emotions
Can these emotional thoughts become exaggerated?
What is the impact of pain on your emotional response?
Mood, anger, anxiety
Can attitudes affect beliefs about pain?

Readability for Clinician Section of Interview Guide: 6.5 Thanks for taking the time to participate in this study! The information we get from you today will help us develop and validate a pain assessment for individuals currently living with NF1. We want to hear from you because you are the experts helping those with NF. The goal of the study is to create a pain assessment for people with NF1 so that healthcare workers, researchers, and people with NF1 can accurately describe and measure pain symptoms. During the session, we will ask you to talk about the pain caused by NF1. Specifically, we are looking at two different types of pain symptoms that can come from NF1: One that is caused by migraines due to NF1, and one that is caused by tumor pain due to NF1. There will also be an opportunity to discuss any other types of pain caused by NF1. Each is important, and we will ask you to describe the type of pain that your patients have. As a reminder, this session will be audio-recorded to help us when we proofread the text copy of this session. We may also take notes to help us remember important points raised that we would want to follow up on. I’d like to stress that the potential benefits of creating an NF1-focused pain measure will only be as good as the information we collect, so it’s important for our discussion to be as open and honest as possible. This will help us understand what we will need to do to create a useful NF1-focused pain measure. Do you have any questions before we start? I am now going to start recording the session.
DOMAIN 1: Assessing NF1 pain experience. Pain is an unpleasant sensation that can affect multiple areas of the body and one’s ability to function. The emotions associated with that pain can also affect individuals’ mood and stress levels. To start, we want to understand your experience treating NF1 pain.
What words do your patients use to describe their NF1 pain?
Burning, stabbing, throbbing
What are your thoughts on the importance of your patients using categories to describe their NF1 pain symptoms?
What other things can occur with/accompany their pain?
Fast heart rate or sweating?
Emotional changes? Changes in function (e.g., ability to move, work, or complete daily living activities)?
Do your patients track their NF1 pain, and if so, how?
Can you tell me how they measure their pain symptoms?
What do they track?
Length, frequency, inter-response time, and cumulation of all?
How does NF1 pain impact their life?
Does it limit your patient’s ability to complete day-to-day tasks?
Quality of Life
Physical Functioning
Range of Motion due to tumor vs. pain
Pain Behavior
Which feature is most important to measure when evaluating NF1 pain symptoms?
Severity
Interference
Quality of life
Normal role functioning
Physical functioning
Emotional wellbeing
DOMAIN 2: How NF1-associated chronic pain can affect other aspects of patients’ lives? In this next section, we’d like to understand the impact of NF1 pain and how it might affect patients in other ways beyond just the pain sensation.
How do they cope with their pain?
What kinds of things do patients do to cope with their pain?
What strategies have they used to cope with their pain?
What strategies have they used to cope with their pain?
Discuss the impact of pain on emotions
Can these emotional thoughts become exaggerated?
What is the impact of pain on their emotional response?
Mood, anger, anxiety
Can attitudes affect beliefs about pain?
How does NF1 pain make your patients respond emotionally?
DOMAIN 3: Opinions about currently available pain measures
What do you think of the current assessments for NF1-associated pain?
Are there merits of the current assessments in your opinion?
If so, what are they?
Are there any demerits of the current assessments in your opinion?
If so, what are they?
What would you like to have measured for pain that is not being measured for pain?
How well do these current assessments evaluate change in NF1-associated pain?
How frequently are these measures needed to get a picture of pain over time?
Is there an impact (normal vs elevated?)
Beyond measuring the physical manifestations of pain, how important is it to understand and measure other NF1 manifestations of pain (e.g., mood, sleep, executive function)?
What is it that makes measuring these covariates important to you in your practice?
DOMAIN 4: Opinions about the uniqueness of NF1-associated pain
a. Do you believe there are NF1-associated pain features that are totally unique to NF1?
In what way?
If so, how?
Would you like to add anything else that you feel we should know in developing an NF1-associated pain measure?
